# Combinatorial Anti-Cancer Effect of Polypurine Reverse Hoogsteen Hairpins against *KRAS* and *MYC* Targeting in Prostate and Pancreatic Cancer Cell Lines

**DOI:** 10.3390/genes15101332

**Published:** 2024-10-16

**Authors:** Simonas Valiuska, Kayla K. Elder, Steven J. McKay, Carlos J. Ciudad, Véronique Noé, Tracy A. Brooks

**Affiliations:** 1 Department of Biochemistry and Physiology, School of Pharmacy and Food Sciences, University of Barcelona (UB), 08028 Barcelona, Spain; simonasvaliuska@ub.edu (S.V.); cciudad@ub.edu (C.J.C.);; 2 Instituto de Nanociencia y Nanotecnología (IN2UB), University of Barcelona (UB), 08028 Barcelona, Spain; 3 Department of Pharmaceutical Sciences, School of Pharmacy and Pharmaceutical Sciences, Binghamton University, Binghamton, NY 13902, USA; kelder1@binghamton.edu (K.K.E.); smckay3@binghamton.edu (S.J.M.)

**Keywords:** KRAS, MYC, PPRH, G-quadruplex, prostate cancer, pancreatic cancer

## Abstract

**Introduction**: *KRAS* and *MYC* are proto-oncogenes that are strictly regulated in healthy cells that have key roles in several processes such as cell growth, proliferation, differentiation, or apoptosis. These genes are tightly interconnected, and their dysregulation can lead to cancer progression. We previously individually targeted these oncogenes using Polypurine Reverse Hoogsteen (PPRH) hairpins, mostly targeting the complementary strand of G-quadruplex-forming sequences. We validated them in vitro in different cancer cell lines with deregulated KRAS and/or MYC. In this work we focused on our understanding of the cooperative dynamics between these oncogenes, by investigating the combined impact of PPRHs targeting KRAS and MYC in pancreatic and prostate cancer cells. **Results**: The combinations had a modulatory impact on the expression of both oncogenes, with transcriptional and translational downregulation occurring five days post-treatment. Out of the four tested PPRHs, MYC-targeting PPRHs, especially HpMYC-G4-PR-C directed against the promoter, showed a greater cytotoxic and expression modulation effect. When both KRAS- and MYC-targeting PPRHs were applied in combination, a synergistic reduction in cell viability was observed. **Conclusion**: The simultaneous targeting of *KRAS* and *MYC* demonstrates efficacy in gene modulation, thus in decreasing cell proliferation and viability.

## 1. Introduction

The human gene Kirsten rat sarcoma viral oncogene homolog (*KRAS*) encodes a membrane-bound regulatory protein belonging to the RAS family of GTPases. When KRAS is activated, it governs downstream cellular pathways responsible for regulating cell survival, proliferation, and differentiation [1,2,3]. Mutations in KRAS disrupt its normal function, resulting in the constitutive activation of the protein, which is linked to an increased tumorigenicity, and it is associated with unfavorable prognosis in several cancers [4]. In the context of aggressive diseases, KRAS is overexpressed in about 30% of all human cancers including 95% of pancreatic and 45% of colorectal and non-small-cell lung cancer (NSCLC) [5,6]. In 2013, the National Center Institute (NCI) established “The RAS initiative” to mobilize the community involved in the research of cancers driven by the RAS gene family (KRAS, HRAS and NRAS) [7]. Several drug discovery efforts are presently underway, with a focus on targeting distinct mutant KRAS isoforms. Sotorasib, targeting the KRAS^G12C^ mutant protein, obtained FDA approval for the treatment of lung cancer in May 2021 [8]. While this development holds promise for cancers bearing this specific KRAS alteration, it is important to note that the G12C mutation is not the only common mutation, and it does not provide advantages for patients with amplified or other KRAS variants. The most prevalent mutated alleles in patients with pancreatic and colorectal cancers are KRAS^G12D^ and KRAS^G12V^, respectively [9,10,11]. Therefore, the approach towards developing treatments adapted to specific KRAS mutations requires the creation of a diverse array of drugs. Inhibiting transcription in cancers with aberrant KRAS expression has proven to be detrimental to tumor cells, revealing their dependency on KRAS independent of its mutational status and provides a wide scope for therapeutic intervention [12,13,14,15].

c-MYC, hereafter referred to as MYC, is a member of a large family of DNA-binding proteins with a basic helix–loop–helix–loop–helix leucine zipper motif, which also includes L- and N-MYC [16,17]. MYC operates as a transcription factor, participating in multiple signal transduction pathways that trigger cell growth and various cellular processes such as metabolism, differentiation, and apoptosis. MYC is tightly regulated in non-cancer cells and quiescent cells have minimal or hardly detectable levels that increase in response to signals related to growth and development [18]. Notably, MYC regulates the expression of more than 30% of human genes [19,20,21]. Its protein is intrinsically fragile, characterized by general instability and a short half-life, making it susceptible to rapid degradation through the ubiquitin-linked proteasome system. This degradation mechanism serves as a safeguard against excessive MYC activity [22]. However, when any of these regulatory mechanisms malfunction, it can result in the presence of abnormal *MYC* mRNA and/or protein, which can lead to the development of malignancies. Alterations in MYC regulation can also be due to factors such as chromosomal translocations, viral insertions, gene amplification, deletions, insertions, and mutations in cis elements [23]. Deregulated MYC expression acts as a driver of both tumor initiation and maintenance and it is linked to all the defining hallmarks of cancer [24]. MYC is recognized as one of the most frequently amplified oncogenes [25]. About 70% of human cancers, including breast, bone, brain, B-cell lymphoma, colon, cervix, lung, pancreas, and prostate tumors have various MYC alterations that correlate with poor prognosis and increased disease aggressiveness [26,27]. Experimental models demonstrating MYC-associated tumorigenesis suggest that established tumors develop MYC dependence or addiction [20].

Both KRAS and MYC have been categorized as undruggable cancer targets. This may be attributed to their extensive protein–protein interactions, lack of clearly defined or appropriate binding sites, or their intracellular or nuclear localization [28,29]. MYC expression is downstream of KRAS signaling. Consequently, the presence of oncogenic KRAS mutations results in sustained MYC expression [30,31]. KRAS-initiated pro-proliferative signals are potentiated by MYC, facilitating rapid cell cycle progression and resistance to apoptosis. This collaboration often leads to the development of more aggressive and therapy-resistant cancers. In some cancer cells with mutated KRAS it was found that its suppression causes polyubiquitination and proteasomal degradation of the MYC protein [32].

Guanine-rich sequences can adopt unique secondary structures known as G-quadruplexes (G4s). These structures consist of four strands and are characterized by a non-canonical arrangement in which the guanine bases are stacked in a planar pattern held together by Hoogsteen hydrogen bonds, resulting in the formation of a G4 structure [33,34]. They are highly present in the promoters of human genes, modulating their expression through transcription factor binding and interaction with other entities [35,36]. Currently, there are more than 370,000 predicted G4 sequences, typically found in regions such as transcriptional start sites (TSS), telomeres, and sites associated with mitotic and meiotic double-strand breaks [35,37]. G4 structures can also act as barriers to block DNA replication [38] and they can interact with proteins implicated in DNA repair processes [37]. Many oncogene promoters, including *KRAS* and *MYC*, have a greater number of G4 motifs than promoters of regulatory or tumor suppressor genes. Several studies have indicated that alterations in G4 sequences are associated with decreased gene expression [39,40,41].

In this study, we used previously designed Polypurine Reverse Hoogsteen (PPRH) hairpins against *KRAS* and *MYC*, either individually or as combined treatments, to decrease the expression of both oncogenes and provoke cell death in KRAS- and MYC-dependent pancreatic (AsPc-1) and prostate cancer (PC-3) cell lines. PPRHs are unmodified single-stranded DNA molecules made of two polypurine strands in antiparallel orientation connected by a loop of four thymidines. The two strands bind intramolecularly to each other through Hoogsteen bonds. PPRHs are designed to bind to a specific polypyrimidine DNA or RNA sequence of a given gene through Watson–Crick bonds. The binding of a PPRH results in the formation of a DNA triplex structure that provokes the displacement of the polypurine chain in the genomic dsDNA, leading to a decrease in the expression of the target gene [42,43,44]. PPRHs do not need to be designed as a pure homopurine sequence since they can include up to three pyrimidine interruptions. This flexibility allows for the design of PPRHs to target practically any gene in the genome [45]. PPRHs are classified as coding (-C) or template (-T), depending on the strand that they are targeting. Coding PPRHs can interact with DNA and mRNA of the target gene. This interaction can influence splicing or translation processes [43,46]. We have previously used PPRHs to target genes related to cancer such as *mTOR, BCL-2, MDM2, TOP1, MYC* [47], and *HER-2* [48]. We have also designed efficient PPRHs targeting complementary sequences of G4-forming sequences (G4FS) in *TYMS* [49], *KRAS* [50], and *MYC* [51], demonstrating PPRH efficacy in reducing cell viability and gene expression. While there is currently no clinically approved drug that directly inhibits MYC [52] ongoing research is progressing for the development of molecules like Omomyc [53].

## 2. Materials and Methods

### 2.1. Polypurine Reverse Hoogsteen Oligonucleotides

**Figure 1:**
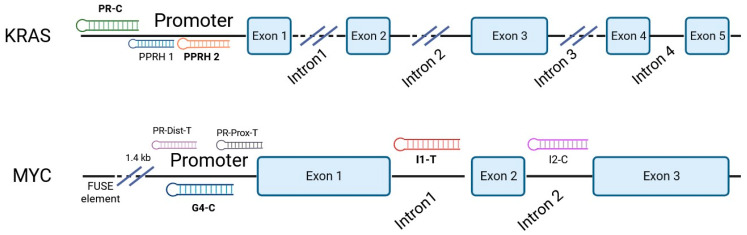
Schematic representation of *KRAS* and *MYC* gene structures and the location of targeting PPRHs. The selected PPRHs, PR-C, PPRH 2, G4-C, and I1-T, for individual and combinatorial treatments are marked in bold.

Previously designed PPRHs against *KRAS* [50] and *MYC* [51] were used throughout this work. Figure 1 provides a schematic representation of *KRAS* and *MYC* gene structures and the location of these PPRHs. Table 1 provides the sequences and names of the oligonucleotides utilized in this study. PPRHs were synthetized as non-modified oligodeoxynucleotides by Merck (Haverhill, UK) or Eurofins Operon (Huntsville, AL, USA), resuspended in sterile Tris-EDTA buffer (10 mM Tris and 1 mM EDTA, pH 8.0) (Sigma-Aldrich, Madrid, Spain) and stored at −20 °C. FAM (MYC-targeting) or HEX (KRAS-targeting) 5′ fluorescent tags were used for live cell imaging.

### 2.2. Cell Culture and Cell Transfection

PC-3 cancer cells were obtained from the Cell Bank resources at the University of Barcelona (UB). AsPc-1 cells were obtained from the American Tissue Culture Collective (ATCC, Manassas, VA, USA). Cell line mutations enumerated in Supplementary Materials [54]. Cells were grown in Ham’s F12 or RPMI-1640 medium, respectively, supplemented with 10% fetal bovine serum (FBS) (GIBCO, Invitrogen, Barcelona, Spain) and maintained in exponential growth conditions at 37 °C in humidified 5% CO_2_ incubator. For cell culture and expansion, a 0.05% trypsin solution (Merck Life Science S.L.U. in Madrid, Spain) was used to dissociate the cells. For all transfections, mixtures were prepared with a fixed amount of 1,2-Dioleoyl-3-trimethylammonium propane (DOTAP; Biontex, Munich, Germany or Fisher Scientific, Waltham, MA, USA) (10 μM) with variable quantities of PPRHs (from 12.5 to 100 nM, or combinations of 25 nM of each PPRH) in a serum-free medium. Following a 20 min incubation at room temperature, the mixture was added to the cells, as described below.

### 2.3. Cellular Viability with MTT Assay

PC-3 cells (10,000 cells per well in a 6-well plate or 2000 cells/well in a 96 well plate) or AsPc-1 (12,500 cells per well in a 96-well plate) were plated and transfected as described in Section 2.2. For cytotoxicity assays, cells were incubated with PPRHs for up to 120 h at 37 °C in a humidified 5% CO_2_ incubator. MTT (Spain group) or MTS (USA group) solution was then added to the wells according to the manufacturer protocols. Absorbance was read at 560 or 490 nM, respectively, on Varioskan microplate readers (Thermo Scientific, Waltham, MA, USA). The cell viability results were expressed as the percentage relative to untreated control cells. The level of synergism between PPRHs was evaluated using CompuSyn software (version 1.0) [55]. The Combination Index (CI) was computed for each combination, and the resultant effect was categorized based on the following values: CI < 0.3 Strong Synergism, CI 0.3–0.7 Synergism, CI 0.7–0.85 Moderate Synergism, CI 0.85–0.90 Slight Synergism, CI 0.90–1.10 Nearly Additive, and CI > 1.10 Antagonism.

### 2.4. Real-Time qPCR

PC-3 or AsPc-1 cells (30,000 or 150–750,000 cells per well, respectively) were initially plated in 6- or 12-well dishes and transfected with the PPRHs using DOTAP the following day. At each indicated time point after transfection, total RNA was extracted with TRIzol^®^ (Life Technologies, Barcelona, Spain) or according to the Roche GeneJet RNA isolation kit (Thermo Fisher, USA) following the manufacturer’s instructions. To assess RNA concentrations, absorbance at 260 nm was measured using a NanoDrop ND-1000 spectrophotometer (Thermo Fisher, Barcelona, Spain).

Up to 1 µg of RNA was reversed transcribed into cDNA. This was accomplished byy employing 125 ng of random hexamers (Roche, Madrid, Spain), 500 µM of each dNTP (Bioline, Barcelona, Spain), 20 units of RNAse inhibitor, and 200 units of Moloney murine leukemia virus reverse transcriptase (the last three components were from Lucigen, Middleton, WI, USA) in the reverse transcriptase buffer. The reaction was incubated at 42 °C for 1 h.

Quantitative PCR (qPCR) was conducted with Taqman probes for *KRAS* (Hs00364282_m1), *MYC* (Hs00153408_m1), and peptidylprolyl isomerase A (*PPIA*) (Hs04194521_s1) (ThermoFisher Scientific, Madrid, Spain) or GAPDH (Hs02758991_g1, ThermoFisher, USA) as the endogenous control. PCR reactions were conducted in a 20 μL volume, which included a 1X TaqMan Universal PCR Mastermix (Applied Biosystems, Madrid, Spain), a 0.5X TaqMan probe, and 3 μL of the cDNA mixture. PCR amplification was performed in a QuantStudio 3 Real-Time PCR System (Applied Biosystems, Barcelona, Spain) or a BioRad CFX96 Real-Time PCR system (BioRad, Hercules, CA, USA). The relative expression of *KRAS* and *MYC* was calculated using the 2^−ΔΔCt^ method. Experiments were carried out in triplicate, and each qPCR reaction was performed with technical duplicates.

### 2.5. Western Blot Analyses

PC-3 cells (60,000 cells per well) were plated in 6-well dishes and transfected with individual PPRHs at 50 nM or in combination of 25 nM each. Total protein extracts were prepared 120 h post-transfection using RIPA buffer (1% Igepal, 0.5% sodium deoxycholate, 0.1% SDS, 150 mM NaCl, 1 mM EDTA, 1 mM PMSF, 10 mM NaF, and 50 mM Tris-HCl, pH 8.0) supplemented with a Protease Inhibitor cocktail (P8340-5ML). All chemicals were purchased from Sigma Aldrich, Madrid, Spain, except for Tris-HCl, which was obtained from PanReac AppliChem, Barcelona, Spain). Cell debris was removed by centrifugation for 10 min at 13,300× *g* and 4 °C. Protein concentrations were determined using the Bio-Rad protein assay based on the Bradford method, employing bovine serum albumin as a standard (Sigma-Aldrich, Madrid, Spain).

Protein extracts were subjected to electrophoresis on 4%/12% SDS-polyacrylamide gels and then transferred to polyvinylidene difluoride membranes (Immobilon P, Milipore, Madrid, Spain) using a semi-dry electroblotter. Membranes were blocked with 5% Blotto, followed by incubation with primary antibodies. For MYC detection, an antibody conjugated to horseradish peroxidase (HRP) was used (dilution 1:1500; ab205818, Abcam, Cambridge, UK). For KRAS detection, a polyclonal rabbit anti human KRAS antibody, conjugated to HRP was used (dilution 1:1200; LS-C211371, Abyntek Biopharma S.L., Biscay, Spain). GAPDH levels were assessed using a primary antibody (dilution 1:500; sc-47724, Santa Cruz Biotechnology, Heidelberg, Germany). Primary antibody incubations were carried out overnight at 4 °C with gentle agitation. For GAPDH, a secondary horseradish peroxidase-conjugated anti-mouse antibody was used (dilution 1:1500, sc-516102, Santa Cruz Biotechnology, Heidelberg, Germany).

KRAS, MYC and GAPDH protein levels were detected using enhanced chemiluminescence (ECL) in accordance with the manufacturer’s instructions (Amersham, Arlington Heights, IL, USA). Protein bands were visualized using the ImageQuant LAS 4000 mini-imager (GE Healthcare, Barcelona, Spain), and quantification was performed using the ImageQuant 10.1 software.

### 2.6. Live Cell Imaging, Confluence, and Cell Cycle Analysis

AsPc-1 cells were plated in 12-well tissue culture treated plates and transfected as described in Section 2.4. Fluorescently labeled PPRHs, as described in Section 2.1, were used. Treated cells were incubated for 120 h in an in-incubator CellLink by Cell Cyte (San Diego, CA, USA) with brightfield, green, and red fluorescence images being recorded throughout the experiment. CELLCYTE Studio, version 2.6.0, software was utilized to evaluate percent confluency as a direct measurement of cell growth for at least two independent experiments. Percent confluency and merged images were extracted from the data for evaluation.

Cells were collected120 h post incubation, washed with PBS and lysed in 70% ethanol at 4 °C until use. Cells were washed in PBS, treated with RNAse A and the DNA was stained with 50 μg/mL propidium iodide solution (in PBS) for 10 min at room temperature before analysis on an BD Accuri C6 plus flow cytometer (Becton Dickinson, Franklin Lakes, NJ, USA). Data were gated for live cells with a 1:1 forward and side scatter profile, and cells with <2n DNA were measured as the sub-G1, ~2n DNA as the G0/G1, 2n < x < 4n as the S, and 4n DNA content as the G2/M phase of the cell cycle. Experiments were performed in duplicate and changes in percent population across treatment groups was compared in GraphPad Prism software (GraphPad, Boston, MA, USA).

### 2.7. Statistical Analyses

Statistical analyses were conducted utilizing GraphPad Prism 10. The data represent the mean value along with the standard error of the mean (SEM) from a minimum of three independent experiments. Statistical significance levels were indicated as described in the figure legends.

## 3. Results

### 3.1. PPRH Selection and Individual and Combinatorial Effect of PPRHs in PC-3 Cell Viability

**Figure 2:**
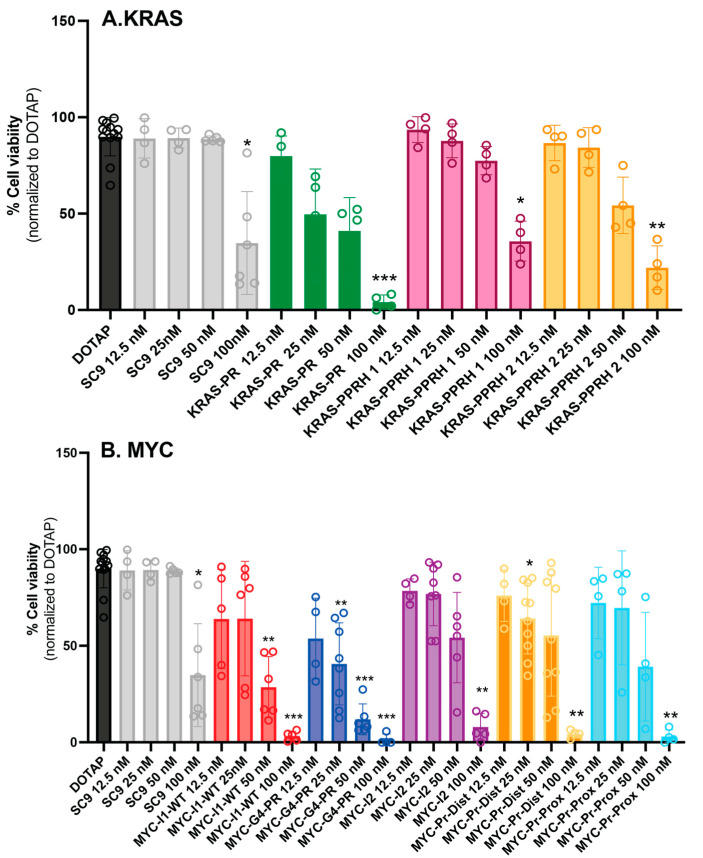
Dose–response results for (**A**) KRAS- and (**B**) MYC-targeting PPRHs on the viability of PC-3 cancer cell line. Cells were treated with doses ranging from 12.5 to 100 nM. Experiments were performed three times with internal duplicates. Statistical significance was determined using a one-way ANOVA with Dunnett’s multiple comparison test comparing groups against DOTAP (* *p* < 0.05, ** *p* < 0.01, *** *p* < 0.001, versus DOTAP).

We performed a dose–response study using previously designed PPRHs against *KRAS* and *MYC* in the prostate cancer cell line PC-3 (Figure 2) [50,51]. Cells were transfected with a mixture of 10 μM DOTAP and a range of concentrations between 12.5 and 100 nM of PPRHs targeting *KRAS* and *MYC* to determine their effects 120 h after transfection. The *MYC* PPRHs assayed were HpMYC-PR-G4-C (G4-C), HpMYC-I1-T (I1-T), HpMYC-I2-C, HpMYC-PR-Prox-T, and HpMYC-PR-Dist-T, as previously published [50,51]. Notably, the dose–response study was expanded in the current study to include 12.5 nM PPRH. We also tested the promoter *KRAS*-targeting PPRHs HpKRAS-PR-C (PR-C), HpKRAS-PR-EF-C (PPRH 1), and HpKRAS-PR-BC-C (PPRH 2). As a negative control we used a scrambled PPRH, Hp-Sc9 (SC9). From the dose–response results (Figure 2), we selected two of the most efficient PPRHs for each gene, G4-C and I1-T for MYC-targeting PPRHs and PR-C and PPRH 2 for KRAS-targeting PPRHs; these PPRHs were also the most effective against the pancreatic cancer cell line, AsPc-1 [50,51].

**Figure 3:**
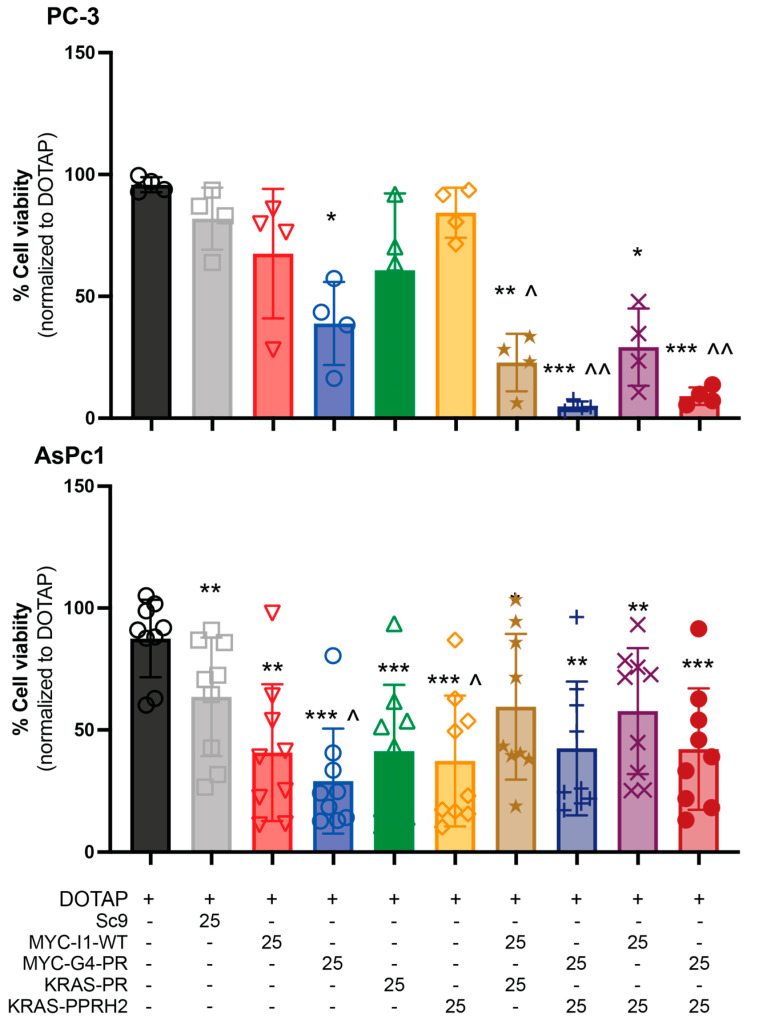
Effect of MYC- (I1-T and G4-C) and KRAS-targeting (PR-C and PPRH 2) PPRHs on the viability of PC-3 (**top**) and AsPc-1 (**bottom**) cell lines. Cells were treated with 25 nM PPRH alone or in combination, as indicated, and incubated for 120 h. Experiments were performed three times with internal duplicates. Statistical significance was determined using a one-way ANOVA with Dunnett’s multiple comparison test comparing groups against DOTAP (* *p* < 0.05, ** *p* < 0.01, *** *p* < 0.001 versus DOTAP control and ^ *p* < 0.05, ^^ *p* < 0.01, versus Sc9 control).

The PPRHs tested in combinations targeting both *MYC* and *KRAS* were G4-C or I1-T for *MYC* and PPRH2 or PR for *KRAS*, in both PC-3 and the previously examined AsPc-1 pancreatic cancer cell lines at a dose of 25 nM per PPRH. This dose was chosen as one that is not too lethal for either cell line, allowing examination of target interaction ranges from synergistic to antagonistic (Figure 3). The PPRH’s effects on cell viability were compared to those obtained after incubating with DOTAP only. SC9 did not have any significant effects on cell viability in the PC-3 cells, but did in the AsPc-1 cells, as previously noted [50,51]. In PC-3 cells, G4-C was the most effective monotherapy, while each therapy was significantly effective at decreasing viability in AsPc-1 cells. Interestingly, the efficacy of G4-C and PPRH2 was significantly greater than the Sc9 control in the pancreatic cancer cell line, indicating they are particularly susceptible to G4-related transcriptional modulation. All combination therapies were effective at significantly lowering cell viability in the AsPc-1 cell line; however, no benefit was noted from the combined targeting of *MYC* and *KRAS* simultaneously. In contrast, all *KRAS-* and *MYC*-targeting PPRH combinations in the PC-3 cells showed a greater efficacy in reducing cell viability. Combinations with G4-C were particularly effective, in agreement with the lone activity of the G4-C PPRH.

Combination Indices (CI’s) were calculated for the PPRH combinations. No synergy was noted with AsPc-1 cells when modulating *MYC* and *KRAS* together. This is in alignment with published works showing that the modulation of MYC expression or KRAS expression are effective in inhibiting the growth of these cells and that the modulation occurs in a single pathway [50,51]. In contrast, CI evaluation showed synergism for PC-3 cells for all combinations at 25 nM, except for the combinations of I1-T and PR-C that demonstrated only a slight synergism (Table 2). Among the four PPRHs, G4-C had the most significant impact on cell viability when used in both individual and combined treatments in both cell lines.

### 3.2. Chemosensitization of Monotherapeutic and Combination PPRHs in PC-3 and AsPc-1 Cells

**Figure 4:**
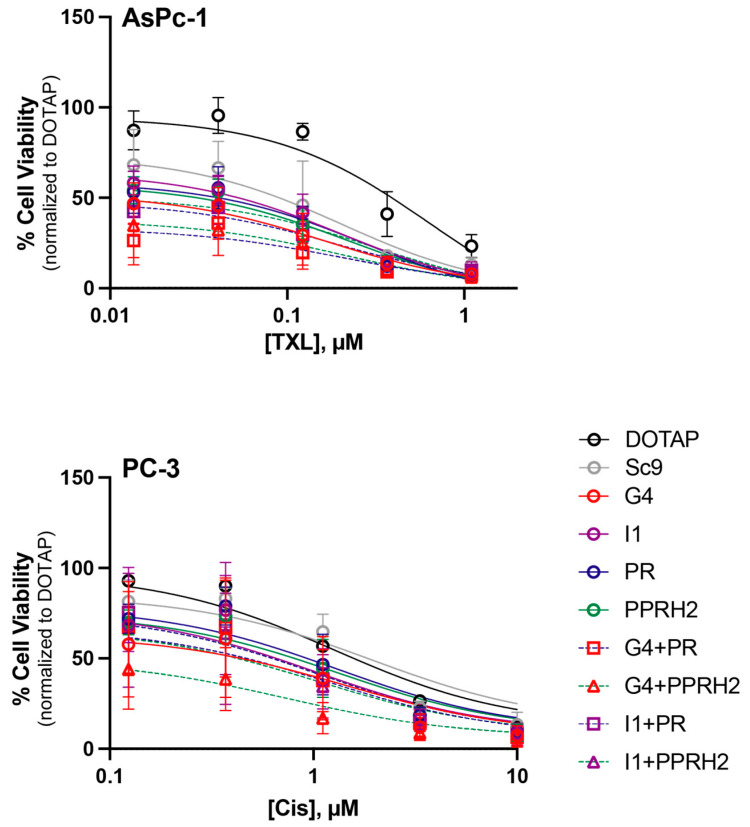
Effect of MYC- (I1-T and/or G4-C) and KRAS-targeting (PR-C and/or PPRH 2) PPRHs on the viability and chemosensitivity of AsPc-1 (**top**) and PC-3 (**bottom**) cell lines to paclitaxel (TXL) or cisplatin (CIS), respectively. Experiments were performed in triplicate.

The potential of the PPRHs to modulate the toxicity of standard-of-care chemotherapeutic agents used in the treatment of prostate and pancreatic cancer was examined. In particular, the toxicity of cisplatin (for prostate cancer) and paclitaxel (for pancreatic cancer) was examined in the presence of up to 25 nM of each PPRH alone or in combination (Figure 4, Table 3). For both cell lines, the PPRHs demonstrated at least additive effects alongside the standard chemotherapies, and some of the combinations demonstrated a sensitization towards the toxic drugs. An IC_50_ of 1.7 μM was observed for cisplatin alone in PC-3 cells, which was maintained between 1.3 and 2.4 μM for most of the PPRHs and PPRH combinations. Notably, the PC-3 cells were sensitized to cisplatin (IC_50_ reduction to 0.9 μM) in the presence of G4-C and PPRH2 combined at 25 nM each. The IC_50_ of paclitaxel was noted to be 0.3 μM in AsPc-1 cells. The addition of 12.5 nM PPRH or PPRH combinations decreased the IC_50_ of paclitaxel for most of the PPRHs and PPRH combinations. While no PPRH or PPRH combination was significantly more or less effective at improving the responsiveness to paclitaxel, lower IC_50_s were noted in combinations of paclitaxel plus a MYC targeting PPRH (average IC_50_ = 0.18, versus 0.25, μM for KRAS targeting PPRH combinations. Overall, the lowest IC_50_ was observed with G4-C + paclitaxel at 0.1675 μM.

### 3.3. Individual and Combinatorial Effects of PPRHs on KRAS and MYC mRNA and Protein Levels

**Figure 5:**
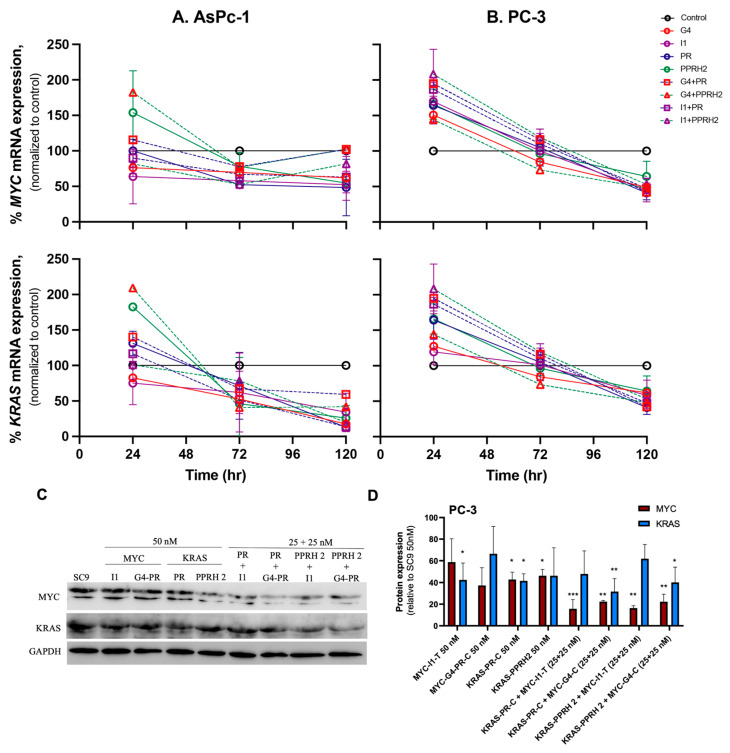
Effect of KRA-S and MYC-targeting PPRHs, individually and in combination, on KRAS and MYC regulation in AsPc-1 and PC-3 cancer cells. Time-course of the PPRHs effect was monitored between 24 and 120 h in AsPc-1 (**A**) and PC-3 (**B**) cancer cells on *MYC* and *KRAS* mRNA levels, expressed relative to SC9 at 50 nM. (**C**) Representative blot of KRAS and MYC protein levels normalized to GAPDH. KRAS and MYC protein levels were monitored 120 h after transfection. (**D**) Quantitation of MYC (red) and KRAS (blue) protein levels. Experiments were performed in triplicate. Statistical significance for each PPRH was determined compared to SC9 control at 50 nM and using one-way ANOVA with post hoc Dunnett analyses (* *p* < 0.05, ** *p* < 0.01, *** *p* < 0.001).

To evaluate the effect of PPRHs on *KRAS* and *MYC* mRNA levels, we kept a fixed final concentration of 50 nM, transfecting the PPRHs either alone or in combinations of 25 nM each, and we performed time-course experiments between 24 and 120 h (Figure 5A,B). We compared the effects relative to SC9 at 50 nM and observed similar mRNA modulation for both oncogenes. The AsPc-1 cells revealed a time-dependent change in gene expression as well, with increased expression at 24 h and maximal decreases of up to 50 and 90% seen in *MYC* and *KRAS* mRNA expression at 120 h post-transfection, respectively (Figure 5A). In the PC-3 cells, both *MYC* and *KRAS* mRNA expression initially increased, reaching a peak around 24 h after transfection, before their expression started decreasing up to 72 h after transfection, reaching the lowest point at 120 h. *KRAS* mRNA expression presented a more subtle increase at early times, reaching a maximum of 150% when transfected with G4-C and PR-C, although no statistically significant results were noted. However, at 120 h after transfection, *KRAS* transcription expression decreased by at least 25% in all cases and a remarkable 50% when G4-C and PR-C were combined. At 120 h, all the PPRH treatments significantly reduced *KRAS* mRNA expression except for PPRH2 (Figure 5B). The modulation of transcription early in the experiment for *MYC* mRNA was more pronounced, increasing 150% in most cases and more than 200% with the combination of I1-T and PPRH2 at 24 h. All PPRH treatments significantly increased *MYC* mRNA expression early in the experiment, except for G4-C and the combination of G4-C with PPRH2. Five days after transfection, all PPRHs reduced *MYC* mRNA levels by more than 50% in all cases except for PPRH2 and the combination of I1-T with PPRH2, which reduced *MYC* mRNA by 30 and 40%, respectively (Figure 5B).

After analyzing the modulatory effects of PPRHs on mRNA levels in time-course experiments, we wanted to study their effects in KRAS and MYC translation at 120 h, when mRNA expression upon PPRH transfection reached its lowest point. We performed Western blot analyses in the same conditions as the RT-qPCR experiments. This included the use of PPRHs individually at 50 nM, and combinations of 25 nM of MYC-targeting PPRHs with 25 nM of a KRAS-targeting PPRH (Figure 5C) and compared the results to SC9. The reduction in KRAS and MYC expression was qualitatively assessed and quantified using the ImageQuant software TL 10.1 and normalized to GAPDH. MYC and KRAS expression were both reduced upon individual or combinatorial transfection of the PPRHs. For KRAS, individual treatments with *MYC*-targeting PPRHs increased protein expression, compared to *KRAS*-targeting PPRHs. Out of the four possible combinations, those with G4-C were the most potent in KRAS downregulation, with a reduction of 65% when combined with PR-C and a reduction of 60% when combined with PPRH2. The reduction in MYC protein expression was stronger than in KRAS, and generally, similar changes in protein levels to those previously determined for its mRNA 120 h after transfection were observed. All of the individual PPRHs reduced MYC protein levels by more than 40%, whereas the *KRAS*-targeting PPRHs, PR-C and PPRH2, reduced them by 60 and 55%, respectively. Although the reduction in MYC protein levels by MYC-targeting PPRHs was not statistically significant, I1-C and G4-C reduced MYC levels by 42 and 62%, respectively. Generally, MYC protein decreases correlated with the observed reduction in PC-3 cell viability, where all four combinations reduced MYC protein expression by 80% or more. I1-T combinations with PR-C and PPRH2 reduced protein levels by 90 and 85%, respectively (Figure 5C).

### 3.4. PPRH Cell Penetrance, Effect on Growth and the Cell Cycle

**Figure 6:**
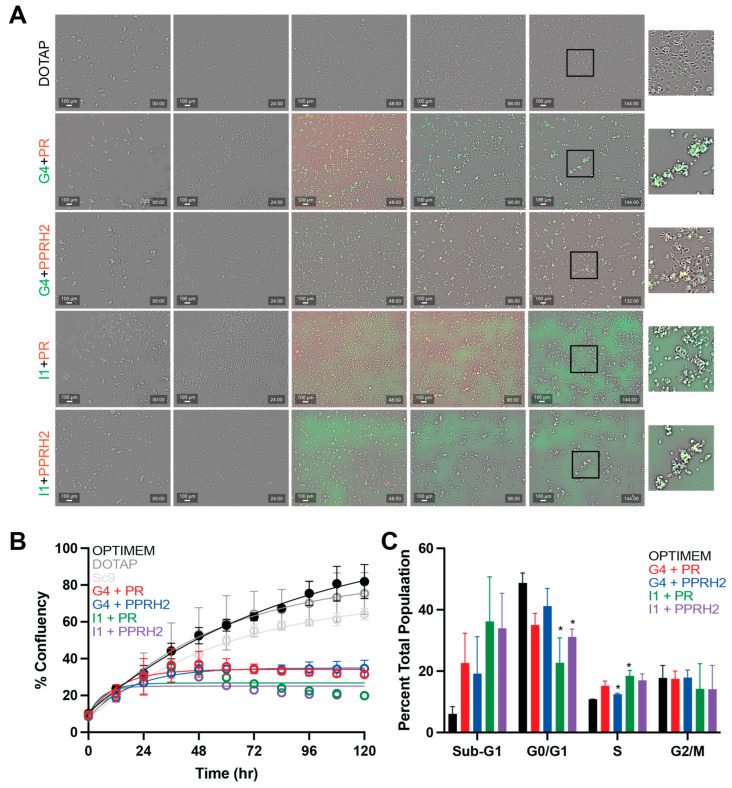
Uptake and outgrowth and cell cycle effects of combined KRAS- and MYC-targeting PPRHs in AsPc-1 pancreatic cancer cells. (**A**) FAM- and HEX-labeled MYC- and KRAS-targeting PPRHs, respectively, were co-transfected into AsPc-1 cells and imaged over time. Transfection occurred 24 h post-plating, and was monitored for a total of 120 h of treatment. Smaller images in the 6th column are zoomed in on the boxed insets in column 5. Yellow fluorescence in all combinations indicates that the indicated MYC- and KRAS-targeting PPRHs were taken up into the cells. (**B**) Confluency was determined from images obtained every 12 h post-transfection over two independent experiments and was compared over time as a measure of cell outgrowth. All combinations demonstrated significant decreases in confluency over time as early as 60 h post-transfection, as determined by two-way ANOVA, and compared to all controls (OptiMEM, DOTAP and Sc9). (**C**) Flow cytometric analysis of DNA, as stained by PI, was used to demonstrate progression through the cell cycle 120 h post-transfection in the indicated combinations. * *p* < 0.05 versus control.

Fluorescently tagged PPRHs were transfected into AsPc-1 cells and an in-incubator live cell imager was used to qualitatively evaluate differential uptake between the FAM (green)-labeled MYC-targeting PPRHs and the HEX (red)-labeled KRAS-targeting PPRHs. Images were taken on a routine basis, and brightfield images were also used to quantitatively determine changes in cell confluency per well, analogous to cell outgrowth over time. At 120 h, cells were collected and analyzed for DNA content and cell cycle progression as well (Figure 6).

Qualitatively, AsPc-1 cells with all MYC- and KRAS-targeting combinations can be visualized as undergoing co-transfection (as noted with apparent yellow fluorescence from the overlap of FAM and HEX dyes). The MYC-I1 and KRAS-PR PPRHs generally presented a more diffuse background at all times points, but no more uptake versus the MYC-G4 or KRAS-PPRH2 PPRHs was noted. PPRHs were added at the 24 h image time point (one-day post-seeding), and a discernable uptake of the PPRHs was noted within the first 24 h post-transfection (Figure 6A). Confluency was evaluated from images taken every 12 h, and all PPRH combinations demonstrate evident and significant effects on overall cell outgrowth (Figure 6B). Changes in growth rates became significant (as determined by a Two-Way ANOVA) within 60 h post treatment, maintaining a low outgrowth out until 120 h post-transfection. The timeline of changes in growth rates coincides with the decrease in target mRNA transcription (Figure 5A) as well.

Changes in cell cycle progression were also examined120h post-transfection, as entrance into and progression through the cell cycle is both initiated by KRAS and facilitated by MYC. Increased sub-G1 populations were appreciated from 6 ± 2.3% in control conditions up to 20.9 ± 8.3 and 35 ± 15.4% for G4 and I1-containing combinations, respectively. This correlates with the changes in cell viability and outgrowth reported throughout the manuscript. Additionally, decreases in the G0/G1 population were noted, which were significant for the I1-containing combination of PPRHs from 48.7 ± 3.2% to 22.7 ± 8.2 and 31.1 ± 2.6% for I1 + PR or PPRH2, respectively. The S-phase populations also increased significantly from 10.8 ± 0.0% to 12.5 ± 0.3 and 18.4 ± 1.8% for the G4+PPRH2 and I1 + PR combinations, respectively. Overall, the combination of I1 + PR demonstrated the most notable and significant changes in cell outgrowth and cell cycle progression amongst the combinations.

## 4. Discussion

Our previous works validated the efficacy of *KRAS-* and *MYC*-targeting PPRHs, which provoked gene silencing and resulted in the death of cancer cells with deregulated *KRAS* and/or *MYC* [50,51]. In this present work, we studied the response to individual and combinatorial *MYC* and *KRAS* targeting PPRHs in KRAS and MYC-deregulated, dependent, and sensitive prostate PC-3 and pancreatic AsPc-1 cancer cells. To do so, we selected four of the most effective PPRHs, two targeting MYC (I1-T and G4-C) and two against KRAS (PR-C and PPRH 2). The combinations of PPRHs showed synergism either with each other or with the standard-of-care chemotherapy technique, demonstrating the potential of combinations of PPRHs against different oncogenes at very low concentrations. The mRNA levels of both oncogenes were increased in the early hours post-transfection followed by the largest reduction five days after PPRH incubation. We hypothesize that an early increase in mRNA levels for both oncogenes is caused by a compensatory effect of increasing transcription after the blockage of mRNA expression upon PPRHs binding to their target regions. The reductions observed in *KRAS* and *MYC* mRNA expression demonstrate the intrinsic relationship between both oncogenes. Changes in cell outgrowth and cell cycle progression were also observed, in agreement with the effects of PPRH combinations on cell viability and mRNA regulation.

One interesting finding in the current work was that the targeting of *MYC* with PPRHs mediated the reduction in *KRAS* mRNA and protein expression. According to some reports, MYC is required for mutant-KRAS-driven tumor initiation and progression, and MYC inhibition has been demonstrated to impair the growth of pancreatic cancer [32,51,56]. Since MYC is a downstream KRAS effector, we expected more relevant effects on *MYC* mRNA and protein levels when targeting *KRAS* with individual PPRHs and combinations. This does not seem to be the case for mRNA levels, but it does for protein. In PC-3 prostate cancer cells, MYC expression was shown to be more sensitive to PPRHs, resulting in a more pronounced downregulation compared to KRAS, whereas the reverse was true in KRAS-mutant and -dependent AsPc-1 pancreatic cancer cells.

Broadly, we observed more marked effects on cytotoxicity, synergy, and chemosensitization with the G4-targeting PPRHs, and more specifically with the *MYC* targeting G4-C PPRH. This PPRH forms a triplex with its target sequence, displacing the complementary strand and allowing the formation of a G-quadruplex structure [51] which enhances *MYC* gene silencing [57]. Previously, our research groups validated the existence of PPRHs targeting the complementary sequences of G4FS to enhance G4 formation in *TYMS* [49], *KRAS* [50], and *MYC* [51]. Additionally, HpMYC-G4-PR-C facilitates G4 structure formation, and it potentially disrupts the binding of Sp1 and CNBP transcription factor. We hypothesize that the main effect observed on cell viability is the result of the specific translation downregulation produced by PPRHs, especially by those targeting *MYC*. Further investigations on an expanded array of KRAS- and/or MYC-dependent cancers are warranted to support the use of PPRHs in combination. Moreover, the introduction of promiscuous G4 stabilizing molecules, such as CX-5461, in combination with PPRHs [47] could further enhance *KRAS* and *MYC* silencing in dependent cancers.

The KRAS and MYC proto-oncogenes have intricate interactions, in which changes in their expression both impact each other and have the potential to initiate tumorigenesis [58]. Current studies propose that MYC plays a crucial role in KRAS-driven cancers [59], although the connection between MYC and drug resistance in these types of cancers remains an unanswered question [60]. MYC has been shown to be a downstream effector of KRAS signaling. In the presence of oncogenic KRAS, MYC will be constitutively expressed [30,31] and stabilized [61], making cells susceptible to DNA damage and apoptosis [62]. Other hypotheses suggest that MYC protein stabilization intensifies the process of tumorigenesis [63]. The strong relationship between KRAS and MYC presents major challenges in the development of targeted therapies, since disrupting one target without affecting the other may trigger compensatory mechanisms that limit the efficacy of treatments. Consequently, strategies that simultaneously target KRAS and MYC have attracted interest as potential solutions. These treatments may hold promise in achieving durable therapeutic responses and overcoming resistance [15,64].

## Figures and Tables

**Table 1:** PPRH sequences used in this study.

Gene	PPRH Name (Location)	Length	Sequence (5′-3′) PPRH + Target
*KRAS*	HpKRAS-PR-C (Promoter)	52	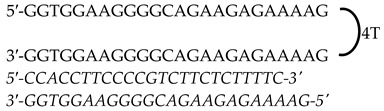
	HpKRAS-PR-EF-C (PPRH 1) (Promoter)	42	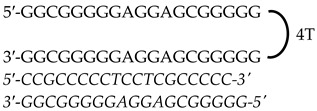
	HpKRAS-PR-BC-C (PPRH 2) (Promoter)	46	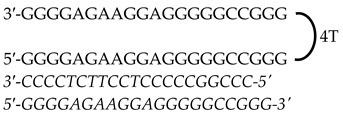
*MYC*	HpMYC-G4-PR-C (Promoter)	67	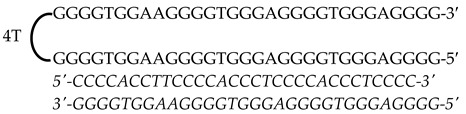
	HpMYC-PR-Distal-T (Promoter)	50	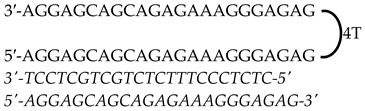
	HpMYC-PR-Prox-T (Promoter)	50	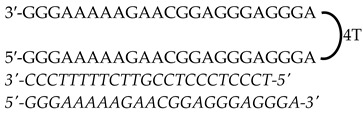
	HpMYC-I1-T (Intron 1)	72	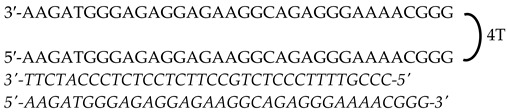
	HpMYC-I2-C (Intron 2)	52	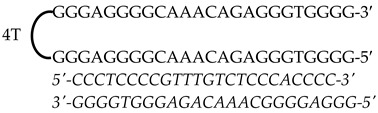

Location, length, and disposition of the PPRH hairpins bound to their target sequences in either *KRAS* or *MYC*.

**Table 2:** Analysis of synergy for the combinations of PPRHs in PC-3 cells.

Dose MYC PPRHs (nM)		Dose KRAS PPRHs (nM)		CI Media	Description
I1-T	G4-C	PR-C	PPRH2		
25	-	25	-	0.88	Slight synergism
25	-	-	25	0.65	Synergism
-	25	25	-	0.57	Synergism
-	25	-	25	0.41	Synergism

**Table 3:** Cytotoxicity of standard-of-care chemotherapy agents paclitaxel (TXL) and cisplatin (CIS) in AsPc-1 and PC-3 cells, respectively, alone and in the presence of PPRHs.

	AsPc-1: TXL IC_50_ (μM)	PC-3: CIS IC_50_ (μM)
DOTAP	0.68	1.734
G4-C	0.1627	1.862
I1-T	0.2016	1.527
PR-C	0.3135	2.041
PPRH2	0.2426	1.856
G4-C+ PR-C	0.2211	1.733
G4-C + PPRH2	0.2739	0.7278
I1-T + PR-C	0.3938	1.422
I1-T + PPRH2	0.2622	1.541

## Data Availability

The data presented in this study are available on request from the corresponding author.

## Supplementary Materials

The following supporting information can be downloaded at: https://www.mdpi.com/article/10.3390/genes15101332/s1, Table S1: Genes mutated in the PC3 cell line, Table S2: Genes mutated in the ASPC1 cell line.
