# Supplementary material for: Combinatorial Anti-Cancer Effect of Polypurine Reverse Hoogsteen Hairpins against KRAS and MYC Targeting in Prostate and Pancreatic Cancer Cell Lines
Source: Genes (Basel). 2024 Oct 16;15(10):1332. doi: 10.3390/genes15101332 (PMC11507358; doi:10.3390/genes15101332)
Supplement: Supplementary file 1 [file genes-15-01332-s001.zip › genes-3128234-supplementary.pdf]

Supplementary Materials

Reference:

Rouillard, A.D.; Gundersen, G.W.; Fernandez, N.F.; Wang, Z.; Monteiro, C.D.; McDermott, M.G.; Ma'ayan, A. The harmonizome: A collection of processed datasets gathered to serve and mine knowledge about genes and proteins. *Database* **2016**, *2016*, pii: baw100.

Table S1: Genes mutated in the **PC3** cell line [1].

Gene Symbol	Gene Name
ABCA3	ATP-binding cassette, sub-family A (ABC1), member 3
ACKR3	atypical chemokine receptor 3
ADAM28	ADAM metallopeptidase domain 28
ADCK3	aarF domain containing kinase 3
ADGRG4	adhesion G protein-coupled receptor G4
AKAP12	A kinase (PRKA) anchor protein 12
AKAP9	A kinase (PRKA) anchor protein 9
ALPK2	alpha-kinase 2
ASTN1	astrotactin 1
AURKC	aurora kinase C
CAMK2A	calcium/calmodulin-dependent protein kinase II alpha
CDK11B	cyclin-dependent kinase 11B
CHIC2	cysteine-rich hydrophobic domain 2
CLTCL1	clathrin, heavy chain-like 1
CREB3L2	cAMP responsive element binding protein 3-like 2
CSF3R	colony stimulating factor 3 receptor (granulocyte)
CTBP2	C-terminal binding protein 2
CXCL12	chemokine (C-X-C motif) ligand 12
CXCR2	chemokine (C-X-C motif) receptor 2
DAB1	Dab, reelin signal transducer, homolog 1 (Drosophila)
DACH1	dachshund family transcription factor 1
DAPK2	death-associated protein kinase 2
EIF4E	eukaryotic translation initiation factor 4E
EPHA6	EPH receptor A6
FGFR1OP	FGFR1 oncogene partner
FLI1	Fli-1 proto-oncogene, ETS transcription factor
FN1	fibronectin 1
GRIA3	glutamate receptor, ionotropic, AMPA 3
GUCY2C	guanylate cyclase 2C
HDLBP	high density lipoprotein binding protein
HIF1A	hypoxia inducible factor 1, alpha subunit
HNF1A	HNF1 homeobox A
HOXA9	homeobox A9

Gene Symbol	Gene Name
IL1A	interleukin 1, alpha
ILK	integrin-linked kinase
IRAK2	interleukin-1 receptor-associated kinase 2
ITPR2	inositol 1,4,5-trisphosphate receptor, type 2
KLK7	kallikrein-related peptidase 7
LETM2	leucine zipper-EF-hand containing transmembrane protein 2
MAF	v-maf avian musculoaponeurotic fibrosarcoma oncogene homolog
MAML3	mastermind-like 3 (Drosophila)
MAP3K1	mitogen-activated protein kinase kinase kinase 1, E3 ubiquitin protein ligase
MAP3K14	mitogen-activated protein kinase kinase kinase 14
MAP4K1	mitogen-activated protein kinase kinase kinase kinase 1
MAPKAPK3	mitogen-activated protein kinase-activated protein kinase 3
MAPKBP1	mitogen-activated protein kinase binding protein 1
MCM3AP-AS1	MCM3AP antisense RNA 1
MSH3	mutS homolog 3
NCAM1	neural cell adhesion molecule 1
NR1H2	nuclear receptor subfamily 1, group H, member 2
OBSCN	obscurin, cytoskeletal calmodulin and titin-interacting RhoGEF
PAX3	paired box 3
PCDH15	protocadherin-related 15
PDE4DIP	phosphodiesterase 4D interacting protein
PGR	progesterone receptor
PI4KB	phosphatidylinositol 4-kinase, catalytic, beta
PIK3C2G	phosphatidylinositol-4-phosphate 3-kinase, catalytic subunit type 2 gamma
PRKCZ	protein kinase C, zeta
PRKDC	protein kinase, DNA-activated, catalytic polypeptide
RECQL4	RecQ protein-like 4
ROBO2	roundabout, axon guidance receptor, homolog 2 (Drosophila)
SEPT9	septin 9
TCF21	transcription factor 21
TFPT	TCF3 (E2A) fusion partner (in childhood Leukemia)
TMEM123	transmembrane protein 123
TP53	tumor protein p53
TP63	tumor protein p63
TRPS1	trichorhinophalangeal syndrome I
TRRAP	transformation/transcription domain-associated protein
TSC2	tuberous sclerosis 2
TTN	titin
VEGFC	vascular endothelial growth factor C

Table S2: Genes mutated in the **ASPC1** cell line [1].

Gene Symbol	Gene Name
ADAM17	ADAM metallopeptidase domain 17
ADAM22	ADAM metallopeptidase domain 22
ADAM28	ADAM metallopeptidase domain 28
ADCK3	aarF domain containing kinase 3
ADGRG4	adhesion G protein-coupled receptor G4
ALPK2	alpha-kinase 2
ARHGAP29	Rho GTPase activating protein 29
BCR	breakpoint cluster region
CHD9	chromodomain helicase DNA binding protein 9
CLTCL1	clathrin, heavy chain-like 1
CSNK1A1L	casein kinase 1, alpha 1-like
DIP2C	DIP2 disco-interacting protein 2 homolog C (Drosophila)
DYRK1A	dual-specificity tyrosine-(Y)-phosphorylation regulated kinase 1A
EIF4E	eukaryotic translation initiation factor 4E
EPHA6	EPH receptor A6
ETV1	ets variant 1
FBXO43	F-box protein 43
FBXW7	F-box and WD repeat domain containing 7, E3 ubiquitin protein ligase
FGFR1OP	FGFR1 oncogene partner
FGFR3	fibroblast growth factor receptor 3
FLI1	Fli-1 proto-oncogene, ETS transcription factor
FLT4	fms-related tyrosine kinase 4
FN1	fibronectin 1
FNBP1	formin binding protein 1
FSCB	fibrous sheath CABYR binding protein
GRIA3	glutamate receptor, ionotropic, AMPA 3
GUCY2C	guanylate cyclase 2C
HNF1A	HNF1 homeobox A
HSP90B1	heat shock protein 90kDa beta (Grp94), member 1
ILK	integrin-linked kinase
INHBA	inhibin, beta A
INSR	insulin receptor
KLK7	kallikrein-related peptidase 7
KMT2A	lysine (K)-specific methyltransferase 2A
KRAS	Kirsten rat sarcoma viral oncogene homolog
KSR2	kinase suppressor of ras 2
LMNB1	lamin B1
LSP1	lymphocyte-specific protein 1
MAML3	mastermind-like 3 (Drosophila)

Gene Symbol	Gene Name
MAP3K2	mitogen-activated protein kinase kinase kinase 2
MAPKBP1	mitogen-activated protein kinase binding protein 1
MCM3AP	minichromosome maintenance complex component 3 associated protein
MYLK	myosin light chain kinase
NCAM1	neural cell adhesion molecule 1
NCOA3	nuclear receptor coactivator 3
NEK3	NIMA-related kinase 3
NFIA	nuclear factor I/A
NOTCH4	notch 4
NR1H2	nuclear receptor subfamily 1, group H, member 2
NUP214	nucleoporin 214kDa
PAK1	p21 protein (Cdc42/Rac)-activated kinase 1
PAX3	paired box 3
PDE4DIP	phosphodiesterase 4D interacting protein
PDGFRA	platelet-derived growth factor receptor, alpha polypeptide
PICALM	phosphatidylinositol binding clathrin assembly protein
PMS1	PMS1 postmeiotic segregation increased 1 (S. cerevisiae)
PPM1E	protein phosphatase, Mg2+/Mn2+ dependent, 1E
PREX2	phosphatidylinositol-3,4,5-trisphosphate-dependent Rac exchange factor 2
PRKCD	protein kinase C, delta
PRKCI	protein kinase C, iota
PRKDC	protein kinase, DNA-activated, catalytic polypeptide
RAPGEF2	Rap guanine nucleotide exchange factor (GEF) 2
SHC1	SHC (Src homology 2 domain containing) transforming protein 1
SMAD4	SMAD family member 4
STAT5A	signal transducer and activator of transcription 5A
STK39	serine threonine kinase 39
STRADA	STE20-related kinase adaptor alpha
TCF21	transcription factor 21
TCL6	T-cell leukemia/lymphoma 6 (non-protein coding)
TMEM123	transmembrane protein 123
TP53	tumor protein p53
TP63	tumor protein p63
TSC2	tuberous sclerosis 2
TTN	titin
VEGFC	vascular endothelial growth factor C
VPS13B	vacuolar protein sorting 13 homolog B (yeast)
ZNF217	zinc finger protein 217
